# The Common Sunstar *Crossaster papposus*—A Neurotoxic Starfish

**DOI:** 10.3390/md19120695

**Published:** 2021-12-07

**Authors:** Karl J. Dean, Ryan P. Alexander, Robert G. Hatfield, Adam M. Lewis, Lewis N. Coates, Tom Collin, Mickael Teixeira Alves, Vanessa Lee, Caroline Daumich, Ruth Hicks, Peter White, Krista M. Thomas, Jim R. Ellis, Andrew D. Turner

**Affiliations:** 1 Centre for Environment Fisheries and Aquaculture Science (CEFAS), Barrack Road, Weymouth DT4 8UB, UK; Ryan.Alexander@cefas.co.uk (R.P.A.); Robert.Harfield@cefas.co.uk (R.G.H.); Adam.Lewis@cefas.co.uk (A.M.L.); Lewis.Coates@cefas.co.uk (L.N.C.); tom.collin@hotmail.be (T.C.); Mickael.teixeiraalves@cefas.co.uk (M.T.A.); Vanessa.Lee@cefas.co.uk (V.L.); caroline.daumich@cefas.co.uk (C.D.); ruth.hicks@cefas.co.uk (R.H.); peter.white@cefas.co.uk (P.W.); andrew.turner@cefas.co.uk (A.D.T.); 2 Department of Chemistry, University of Surrey, Guildford GU2 7XH, UK; 3 Biotoxin Metrology, National Research Council Canada, Halifax, NS B3Z 3H1, Canada; Krista.thomas@nrc-cnrc.gc.ca; 4 Centre for Environment Fisheries and Aquaculture Science (CEFAS), Pakefield Road, Lowestoft NR33 0HT, UK; Jim.Ellis@cefas.co.uk

**Keywords:** benthos, north east Atlantic, sunstars, solasteridae, paralytic shellfish toxins

## Abstract

Saxitoxins (STXs) are a family of potent neurotoxins produced naturally by certain species of phytoplankton and cyanobacteria which are extremely toxic to mammalian nervous systems. The accumulation of STXs in bivalve molluscs can significantly impact animal and human health. Recent work conducted in the North Sea highlighted the widespread presence of various saxitoxins in a range of benthic organisms, with the common sunstar (*Crossaster papposus*) demonstrating high concentrations of saxitoxins. In this study, an extensive sampling program was undertaken across multiple seas surrounding the UK, with 146 starfish and 5 brittlestars of multiple species analysed for STXs. All the common sunstars analysed (*n* > 70) contained quantifiable levels of STXs, with the total concentrations ranging from 99 to 11,245 µg STX eq/kg. The common sunstars were statistically different in terms of toxin loading to all the other starfish species tested. Two distinct toxic profiles were observed in sunstars, a decarbomylsaxitoxin (dcSTX)-dominant profile which encompassed samples from most of the UK coast and an STX and gonyautoxin2 (GTX2) profile from the North Yorkshire coast of England. Compartmentalisation studies demonstrated that the female gonads exhibited the highest toxin concentrations of all the individual organs tested, with concentrations >40,000 µg STX eq/kg in one sample. All the sunstars, male or female, exhibited the presence of STXs in the skin, digestive glands and gonads. This study highlights that the common sunstar ubiquitously contains STXs, independent of the geographical location around the UK and often at concentrations many times higher than the current regulatory limits for STXs in molluscs; therefore, the common sunstar should be considered toxic hereafter.

## 1. Introduction

**Figure 1:**
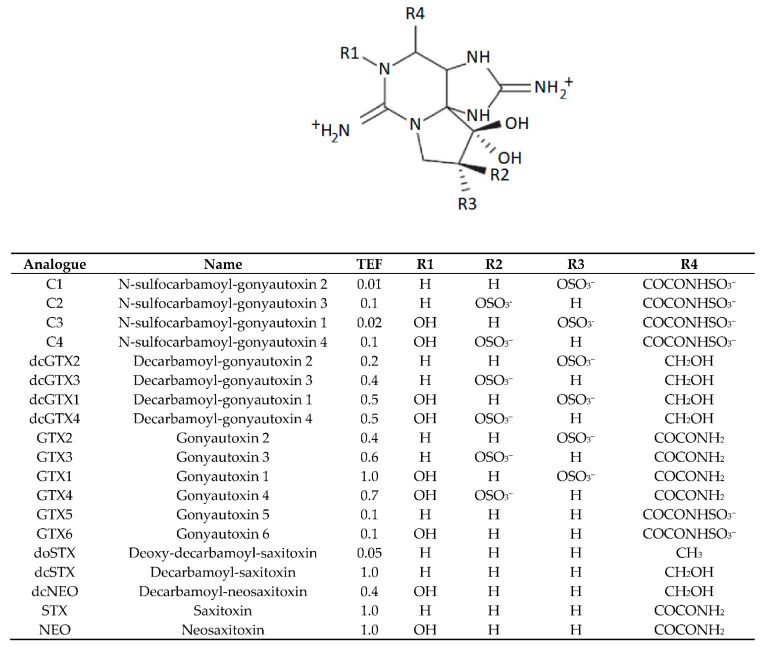
Chemical structures and TEFs (toxin equivalence factors) of the common STXs. TEFs primarily based on EFSA recommendations.

The saxitoxins (STXs) are a group of structurally related neurotoxic alkaloids responsible for the human health syndrome paralytic shellfish poisoning (PSP) [1]. The parent compound saxitoxin (STX), as well as over 50 known analogues, have been described (the common STXs are detailed in Figure 1), all with varying toxicities [2,3]. STXs bind to site one of the voltage-gated Na+ channel, thus stemming the flow of sodium ions into excitable cells. Symptoms include tingling in the extremities, numbness of the lips, vomiting, headaches, ataxia, paralysis and, in severe intoxications, death via respiratory arrest [4]. The toxins are commonly associated with harmful algal blooms of the genera *Alexandrium*, *Gymondinium* and *Pyrodinium* [5,6], as well as some freshwater cyanobacteria [7]. Due to the filter-feeding capacity of bivalve molluscs, the bioaccumulation of STXs into these foodstuffs is a vector of intoxication to humans and animals. To manage the risk to shellfish consumers from PSP, the regulatory testing of bivalve molluscs is a near global requirement, with a maximum permitted level (MPL) of 800 µg STX eq/kg stipulated in EU law [8,9,10].

In the UK, the most common known producers of STXs are the marine dinoflagellate species *Alexandrium catenella* [11,12] (reported as *Alexandrium tamerense* group 1) and *A. minutum* [13]. The common toxin profiles of each *Alexandrium* species are well described in UK shellfish [14], with *A. catenella* from Scotland producing a mixed profile containing gonyautoxins1-4 (GTXs), neosaxitoxin (NEO) and saxitoxin (STX) and *A. minutum* profiles from England and Wales dominated by GTX2&3 and STX. Multiple species within the genus *Alexandrium* are capable of producing a resilient ‘rest’ phase in their life cycle [15], transitioning from the water column into the sediment. These cysts are capable of containing high concentrations of STXs, and consumption of these cysts has been implicated in the accumulation of STXs in shellfish [16,17]. Benthic grazers, such as those that feed on echinoderms, have been previously noted to consume algal cysts, and it is therefore possible that exposure to toxic algal cysts through their natural feeding patterns can lead to the accumulation of STXs [18]. Cyanobacteria also have benthic variants capable of producing STXs [19]; however, these are limited to freshwater or marginal environments, and to the authors’ knowledge, STX-producing cyanobacteria have not been detected in UK water bodies to date. The production of STXs by marine bacteria is still questionable, with many suspected STX producers isolated from the known STX-producing dinoflagellates [20,21] and results generated using non-specific detection methods [22,23]. It has been proposed that marine bacteria are involved in the production of the neurotoxin tetrodotoxin (TTX) in marine organisms, including pufferfish [24] and the starfish *Astropecten polyacanthus* [25]. The biosynthesis of STXs has been mapped in cyanobacteria and dinoflagellates, and the proposed genes attributed to its production have been elucidated [7,26,27,28,29]. The production pathway for these toxins is a multistage synthesis requiring a series of core, regulator, tailoring and transporter genes. The process starts with the sxtA4 gene cluster facilitating the production of a 4-amino-3-oxo-guanidinoheptane intermediate from arginine and ends after a cascade of sxt-gene-controlled reactions with the production of decarbamoylsaxitoxin (dcSTX), which is subsequently transformed into the parent STX after the addition of a carbamoyl group at C-13 (R4 in Figure 1) [26,27].

Traditionally, the detection, accumulation and depuration of STXs has been focused on bivalve molluscs [4,6,14,30,31,32,33,34,35,36,37,38,39], due to their ability to bioaccumulate toxins and their role as an important seafood product. Steadily, research into non-bivalve occurrences has increased understanding, with STXs discovered in many other taxonomic groups, including fish [30,40,41,42,43] and marine mammals [44,45,46,47]. Additionally, multiple investigations into invertebrates have highlighted that STXs are more common in these vectors than previously thought [41,48,49,50,51]. Published literature now provides evidence that STXs are present at multiple trophic levels across wide taxonomic groups. In the winter of 2018, a large winter storm stranded multiple marine organisms along the coast of eastern England. Ingestion of these washed-up organisms resulted in multiple canines falling ill and two fatalities [52]. Subsequent investigation concluded that STXs were the probable cause of death. Surprisingly, the common sunstar (*Crossaster papposus*) exhibited extreme toxicities, which exceeded 14,000 µg STX eq/kg [52], with crustacean and fish samples also accumulating toxins. The presence of STXs in this location at this time of year was unexpected, given that there had been no historical outbreaks in the area [14], no presence of typical algal producers at the time [53] and no accumulation of STXs in the onshore shellfish beds. The toxin profile discovered was also unexpected, with a high percentage of the toxic burden attributed to dcSTX, and STX and gonyautoxin5 (GTX5) also present in lower concentrations. This toxin profile was unlike that of any known algal producer, both domestically and globally. The high proportion of dcSTX suggested a possible enzymatic change, due to the presence of carbomylase and sulfocarbomoylase [29,54,55] or the presence of STX-transforming bacteria [21]. Recent studies have described the accumulation of STXs in a wide range of taxonomic groups across broad areas of the North Sea. In addition, high toxin concentrations exceeding 1000 µg STX eq/kg were quantified in common sunstars [56] sampled from multiple locations. Although sunstars appeared consistently toxic, the toxin profiles were driven by location, with a high dcSTX profile determined in most North Sea locations and an STX and GTX 2 dominated profile discovered off the North Yorkshire coast (North East England). Sunstar toxicity had never been described before these two studies, and STXs have rarely been reported in other starfish species [41,57,58,59,60] (in these cases, starfish toxicity was linked to a causative algal bloom and subsequent predation of intoxicated molluscs, which differs to the previous two studies described here). The source of STXs in sunstars and the wider benthos has yet to be elucidated.

The common sunstar is present around most of the British Isles [61] and has a broader circumboreal distribution [62]. The common sunstar is primarily predatory, feeding on most appropriately sized invertebrates that are available, but it also displays scavenging and cannibalistic feeding behaviours [18,63,64,65,66,67]. Mature sunstars are rarely predated on by other animals, with larger sunstars (e.g., *Solaster* spp.), being its most noted predator [63,66]. Sunstars are known to illicit a strong avoidance response in many organisms [66,68,69], including other starfish species, via physical interaction and distance chemoreception. Sunstars often do not exert enough (or any) force on bivalve molluscs; however, they are still successful mollusc predators [68]. This could imply the possible presence of a toxic aid in their predation mechanics. Although the presence of toxic compounds in starfish has been accepted, its use as a tool for predation has been questioned [70]. The anecdotal death of cats fed with sunstars [65,68] has been previously noted, and a series of biologically active saponins have been derived from sunstars [71].

In two previous studies, all examples of *C. papposus* analysed contained STXs ([52], n = 2, and [56], n = 7), regardless of the location or the time of the year; therefore, there is a potential risk to seafood consumers, as the trophic transfer of STXs through the food chain is common [60,72,73,74]. STXs also have a wide effect on marine organisms [75], such as starfish [60], fish [40,42,76], bivalve molluscs [77,78], gastropods [73] and sea urchins [79]. Exposure to STXs can also affect marine mammals such as whales, seals [44,45] and otters [46], as well as sea birds [80,81,82,83]. This study sought to extensively map out the level of sunstar toxicity around the UK coast and, ultimately, to better understand any geographical or physiological drivers of toxin presence.

## 2. Results

### 2.1. Starfish Toxicity

**Figure 2:**
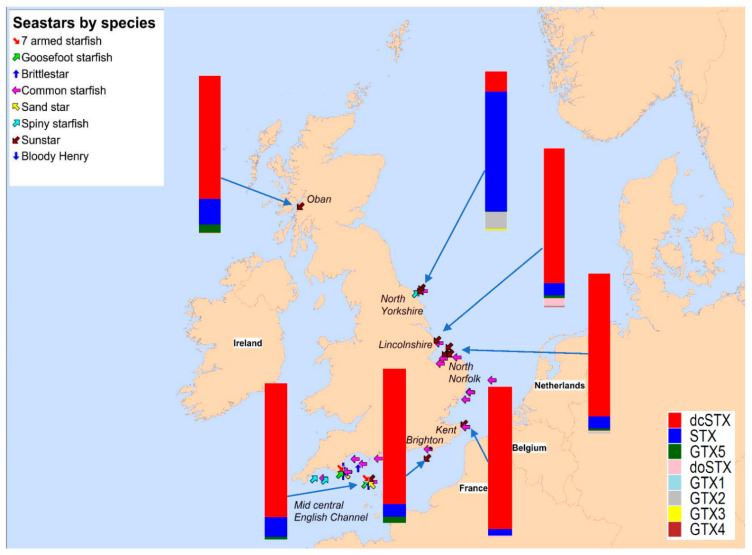
Sampling locations of all starfishes collected, with mean toxic profiles of sunstars at each sampling region expressed as toxin load % in µg STX eq/kg.

**Figure 3:**
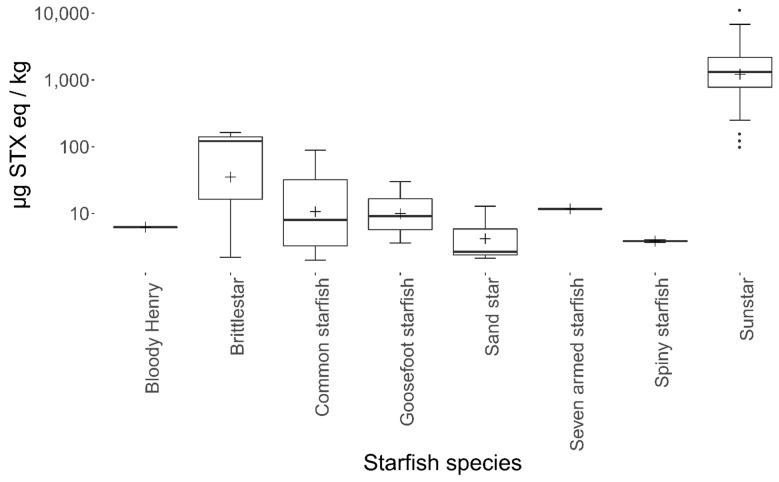
Box-and-whisker plot highlighting species means (cross), 1st and 3rd quartiles, outliers (dots) and interquartile ranges for the starfish and brittlestar species analysed (sample numbers (n) can be found in Table A2).

As the common sunstar *C. papposus* (referred to hereafter as sunstar) was suspected of displaying a consistent presence of STXs, a range of other starfish species were analysed, to act as a control group. Figure 2 summarises the sampling locations of all the starfishes sampled. In total, 151 starfishes were analysed between 2018 and 2021 (Table A1), comprising 73 specimens of sunstars and six other starfish species (n = 73), with one brittlestar species also analysed (n = 5). In sunstars, the presence of STXs was ubiquitous, with a mean total toxin concentration of 1739 µg STX eq/kg (Figure 3 and Table A2). In some cases, extreme toxicity in sunstars was quantified, with a maximum level of 11,245 µg STX eq/kg recorded in one sunstar from north Norfolk.

**Figure 4:**
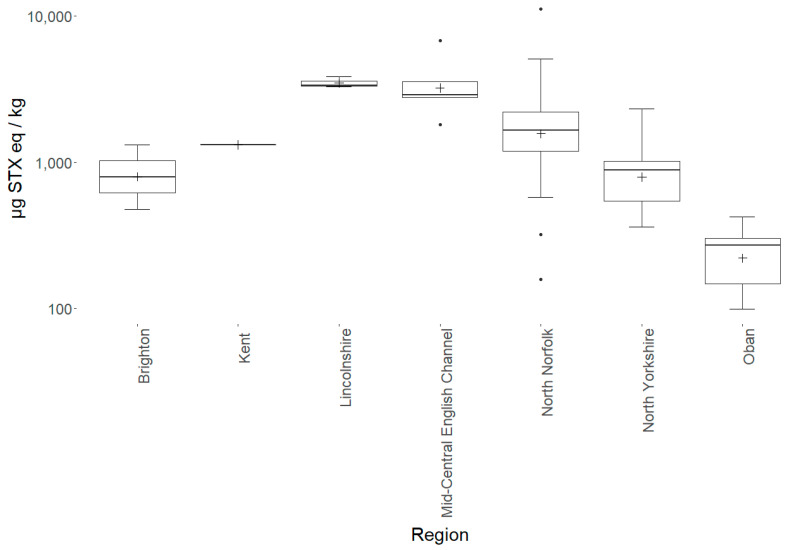
Box-and-whisker plot highlighting sampling region means (cross), 1st and 3rd quartiles, outliers (dots) and interquartile ranges for all sunstars analysed.

Conversely, none of the other starfish species showed any consistent toxicity, with the highest non-sunstar toxin concentration quantified in the brittlestar *Ophiura ophiura* (164 µg STX eq/kg). As some species were under-represented (Table A2), the species were grouped into sunstars and non-sunstars for statistical analysis. The sunstars exhibited higher mean toxicities in comparison with the other starfish species. This was statistically assessed with an ANOVA, which highlighted the species as the most statistically significant factor affecting the toxicity at the 95% significance level (*p* = <2 × 10^−17^). Additionally, the geographic region also had a statistically significant effect on the toxicity (*p* = 3.8 × 10^−8^); however, no statistical effect of starfish diameter or temporal variability was found. A linear mixed-effects model was fitted with the sampling region as a fixed variable and the species as a random variable, which highlighted the mid-central English Channel region as being statistically different to other regions (*p* = 0.0257). A Tukey’s multiple comparison of means test confirmed a significant difference in the toxin levels quantified in samples from the mid-central English Channel compared with those from most other sampling locations. Figure 4 highlights the geographical differences in toxicity across the UK. It should be noted that the sample numbers were low for many regions and most of the sunstars tested were from North Norfolk (n = 40) (see Table A3 for an overview of each region), where the intoxication event in canines occurred, which may have biased the geographical differences and requires further exploration. The interanimal variance in the total toxin concentrations was calculated using 22 sunstars sampled from the Wash in north Norfolk on the same day. The sunstars had a mean of 1558 µg STX eq/kg, a standard deviation of 570 µg STX eq/kg and an RSD of 37%, suggesting moderate inter-animal variability. The RSD of all the sunstars was 89%, showing large variability across the entire population. Therefore, it appeared that factors other than the geographical location may have affected the toxicity, as the sunstars from north Norfolk ranged from 157 to 11,245 µg STX eq/ kg (the low result from Holkham Beach in February 2018 and the high result from the Wash in January 2020). Overall, these data highlight the ubiquitous presence of STXs in sunstars and provide strong evidence that they exhibit STX presence differently to other starfish species. As the LC–MS/MS method utilised for the quantitation of the STXs could quantify the neurotoxin tetrodotoxin (TTX), all the samples were also analysed for the presence of this toxin; however, TTX was not detected in any sample.

### 2.2. Toxin Profiles

**Figure 5:**
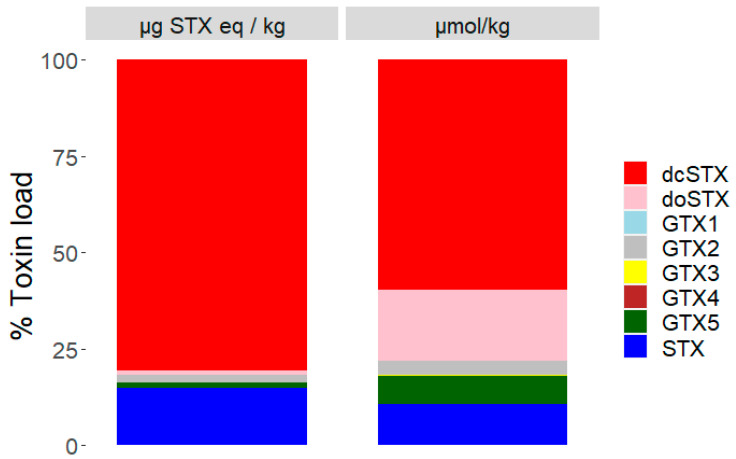
Mean toxic profiles of all sunstars expressed as toxin load % in µg STX eq/kg (**left**) and µmol/kg (**right**).

With the non-sunstar starfish (and brittlestars) containing low or nondetectable levels of STXs, toxic profiles were not generated for them. Analyses of the mean profiles were undertaken for sunstars in terms of both micrograms of STX equivalents per kilogram and micromoles per kilogram (Figure 5). The mean sunstar profile was dominated by dcSTX, with smaller relative contributions from deoxydecarbomyl-STX (doSTX), STX, GTX5 and GTX1–4. Large differences in the toxin proportions of doSTX and GTX5 between the micrograms of STX equivalents per kilogram and micromoles per kilogram profiles were noted as a consequence of the low relative TEF of these toxins. The mean profile from each geographic region (Figure 2) highlighted the differing toxic profiles based on the geographic location. There were two discernible profiles described, one dominated by STX and GTX2 from a relatively small sampling area, specifically, off the North Yorkshire coast, and all other regions exhibiting a dcSTX and STX profile. This did not appear to be a latitudinal-driven phenomenon, as susntars from Oban (NW Scotland) also had a dcSTX-dominated profile. This was confirmed by an ANOVA that demonstrated that the dcSTX load was statistically driven by the sampling region (*p* = <6.9 × 10^−14^), and a Tukey’s multiple comparison of means test corroborated the ANOVA results by highlighting the sunstars from North Yorkshire as having statistically different dcSTX loads to those of most other sampling regions. The dcSTX-dominated profiles varied slightly based on the location. Specifically, the Lincolnshire, north Norfolk and Kent sunstars showed concentrations of doSTX, which was not present in the South Coast sunstars (Brighton and the English Channel); instead, the doSTX portion was replaced with a GTX5 component.

### 2.3. Sunstar Physiological Analysis

**Figure 6:**
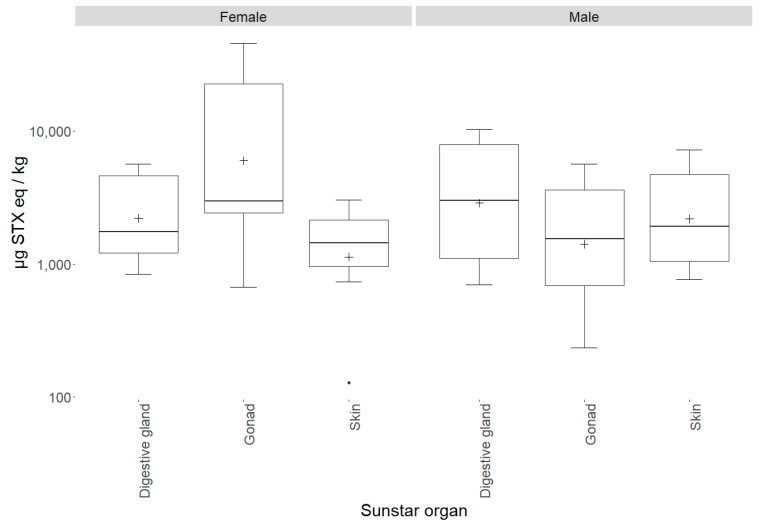
Box-and-whisker plot highlighting individual sunstar organ means for each sex (cross), 1st and 3rd quartiles, outliers (dots) and interquartile ranges.

**Figure 7:**
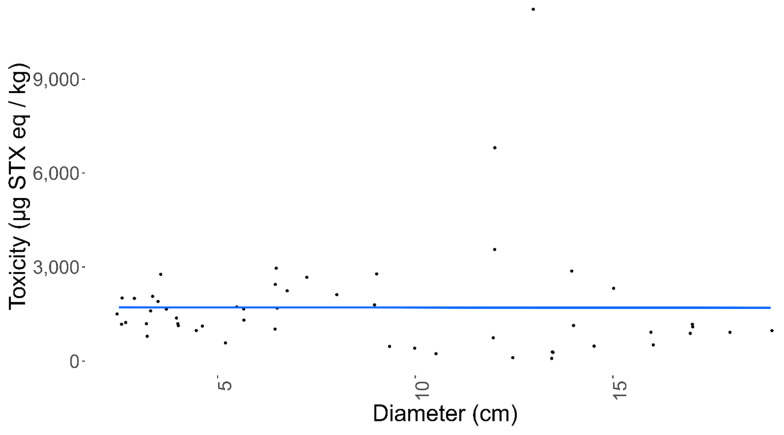
The relationship between the diameter of sunstars and toxicity.

To determine any variability in the STX concentration between the sunstar organs, 13 sunstars from three different batches, each obtained from different sampling locations on different dates, were dissected, and the digestive glands, skin and gonads were removed and tested separately. At this point, the sunstars were sexed visually and, where required, this was confirmed via a histological examination through light microscopy of the gonads. The sunstars that were unable to be sexed or were sexually immature were removed from the analysis. The first batch of sunstars were from North Yorkshire and consisted of three males and two females, and they exhibited the STX- and GTX2-heavy toxin profile. The second batch originated from the Devon coast (Southwest England) and consisted of three males and two females, and the third batch from north Norfolk, which consisted of three females. The sunstars from batches two and three exhibited the dcSTX and STX profile. The highest toxin content (45,766 µg STX eq/kg) was quantified in a female gonad sample, with the female gonads also exhibiting the highest mean toxicity (14,234 µg STX eq/kg) of all the organs analysed (Figure 6, Table A4). In the females, the interorgan variability in toxicity was large, whilst in the males, all the organs appeared to show similar toxicities. An ANOVA assessing the total toxin concentrations in relation to the organisms’ sex, batch and organ type showed that the batch had a statistically significant effect on the toxicity (*p* = <3.53 × 10^−11^). The mean toxicities for all the organs combined for each batch were 1176, 4056 and 12,589 µg STX eq/kg for batches one to three, respectively. Neither the sex nor the organ type showed a significant effect on the toxicity. To remove the effect of interbatch toxicity on identifying whether sex has a statistically significant effect on toxicity in sunstars (batch three heavily skewed the results towards females), a linear mixed-effects model was fitted with the sex as a random variable and the batch as a fixed variable. This highlighted that there was no statistical difference between male and female toxicity (*p* = 0.12). As each sex was not fully represented in all the batches and the batch had a statistically significant effect on the toxicity, the analysis of the organs’ toxicity was carried out separately on each sex. When a linear mixed-effects model was fitted for the female sunstars, using the organ type as a random variable and the batch as a fixed variable, the female gonads showed a weak but statistically significant influence on the toxicity (*p* = 0.02) and a Tukey’s analysis of multiple means highlighted statistically significant differences between the gonads and the skin, and the gonads and the digestive glands (*p* = 0.001 and *p* = 0.033, respectively). The same model was fitted for the male sunstars. This highlighted the gonads as being statistically significantly different to the other organs (*p* = 0.009), with the gonad–digestive gland interaction showing a statistically significant difference (*p* = 0.007), but the gonad–skin interaction demonstrating no statistical difference. In conclusion, sex had no statistically significant effect on the overall toxicity. However, for each sex, the gonads appeared to show different toxicity levels compared to the other organs: in the females this manifested in a higher toxicity, and in the males in a lower toxicity. Although these data offer some weight to the notion that different organs display different toxicities, the sample sizes were small and, as such, drawing definitive conclusions from them is questionable. In order to assess any potential relationship between the organism size and the total toxin levels, the quantified toxicities were compared between the measured sunstar diameters. Figure 7 illustrates the lack of any apparent correlation between the sunstar size and the toxin levels. This was confirmed by the ANOVA described in 2.1, which highlighted no statistical effect between the diameter of a sunstar and the total toxin concentration.

### 2.4. Comparison of Detection Techniques

**Figure A1:**
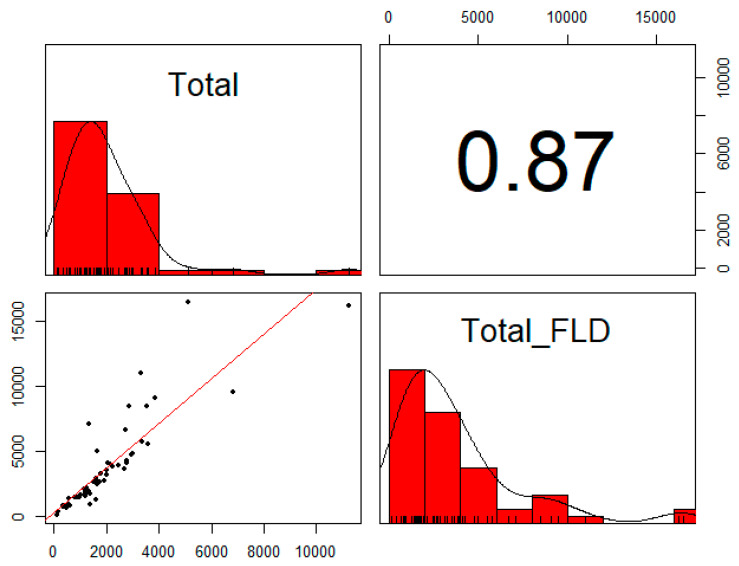
Comparison of LC–FLD and HILIC–MS/MS methods. Correlation coefficient (top right), scatter plot (bottom left) and distributions (HILIC–MS/MS (top left) and LC–FLD (bottom right)). The x axis is HILIC–MS/MS in µg STX eq/kg and y axis is LC–FLD in µg STX eq/kg.

**Figure A2:**
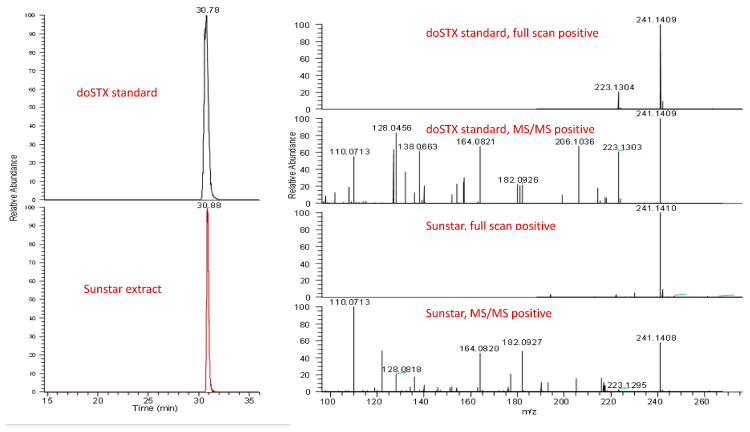
LC–HRMS chromatogram showing accurate mass and fragmentation pattern of doSTX in a standard (not certified) and a sunstar sample.

As there are no formally validated methods for the determination of STXs in starfish tissues, quantitation was performed using two independent methods, the precolumn oxidation and liquid chromatography with fluorescence detection (LC–FLD) method and the tandem mass spectrometry utilising HILIC (HILIC–MS/MS) method (See Figure A1 for a comparison between the methods). There was a good agreement between the two quantitative methods, as the correlation coefficient of the total toxicity was 0.87, with dcSTX, STX and GTX5 having the coefficients of 0.87, 0.91 and 0.55, respectively. A paired Student’s *t*-test confirmed a statistical difference between the two methods as a consequence of the consistent slight overestimation of the LC–FLD method vs. the HILIC–MS/MS method. This was likely due to matrix-related interference during ionisation, as seen in other species [84]. As neither method has been fully validated, it is currently not possible to elucidate which is quantitatively more accurate; as such, the HILIC–MS/MS method was used for the bulk of the analysis to take a conservative approach, and due to its ability to quantify all the toxic epimers individually [10]. Qualitatively, the methods agreed well, with dcSTX, STX and GTX5 always detected in positive samples which exhibited the dcSTX profile. However, doSTX was not detected using the LC–FLD method, although it was detected by HILIC–MS/MS. The preCOX LC–FLD method can detect doSTX [85]; however, due to the rapid chromatographic nature of the LC–FLD method used, it is possible that the doSTX coeluted with the STX, making confirmation by LC–FLD impossible without changing the chromatographic methods. The confirmation of doSTX presence was, however, conducted via an LC–HRMS method. An accurate mass-to-charge-ratio measurement of 241.1413 (∆ = 2 ppm for C_9_H_17_N_6_O_2_^+^) was obtained for the [M + H]^+^ ion of doSTX (SI Ax) (Figure A2). Overall, the methods agreed well enough for good confidence in the HILIC–MS/MS data used for the qualitative and quantitative analysis.

## 3. Discussion

### 3.1. Starfish Toxicity

An extensive sampling and toxicity screening program was conducted in waters around the UK coast to assess the prevalence of STXs in starfish. The data obtained provide strong evidence to support the preliminary hypothesis [52,56] that sunstars ubiquitously contain STXs (n = 71). All the sunstars sampled contained quantifiable concentrations of toxins in all the sampling locations across all the sampling dates. The toxicity in the mid-central English Channel (Figure 2 and Figure 4) was statistically different to those in the other sampling regions; however, due to the random nature of the sampling and the fact that only a small number of sunstars were available from regions other than north Norfolk, drawing conclusions on the geographic variability is difficult. The variability over the entire sunstar dataset was large (RSD 89%); however, the interanimal variability of a subset sampled on the same day from the same location showed an RSD of 37%. This represented a relatively low interanimal variability and was lower than is commonly seen in shellfish [31,86,87,88,89]. Published records in peer-reviewed literature for the accumulation of STXs in starfish are rare [41,57,58,59,60]. In these manuscripts, the accumulation of STXs in starfish was linked to their predation on contaminated bivalves following algal blooms. An analysis of annual phytoplankton results [54,90] showed no correlation between toxic sunstars and the presence of vegetative *Alexandrium* cells in the water columns or contaminated bivalves from routine monitoring points. The only notable occurrence was related to samples collected in Oban in March 2021, when low *Alexandrium* cell counts were detected in the surrounding area. It should be noted that these routine monitoring points do not specifically relate to the sampling points of sunstars in this study, but they could be used as a general indication of the algal presence in the surrounding area. Furthermore, there were many offshore collections of starfish that had no inshore monitoring point in close proximity; therefore, elucidating the presence of causative phytoplankton species at these sampling points was not possible.

Consistent sunstar toxicity is hard to explain, especially compared to other starfish species occupying the same geographical and ecological niches. The vast geographic range of sunstar toxicity also makes the accumulation via an algal cell/cyst route questionable. The accumulation of STXs via trophic transfer by ingesting intoxicated food sources is also unlikely, as sunstars are both scavenger and predatory by nature [65,69]; therefore, their food sources would be expected to be different depending on what is readily available in each location. Conversely, the presence of STXs in sunstars could result from a dietary source, as, although they occupy similar ecological roles to other starfish species, their feeding habits have been shown to be different [66,67,69,91]. However, *C. papposus* often consumes the common starfish *Asterias rubens* as a preference over molluscs or gastropods [18,65,91], and, as the common starfish displayed far fewer STXs (in comparison to the sunstars), sunstars are unlikely to be accumulating STXs via this route. A trophic transfer route of STXs could be explained if sunstars possessed an active storage mechanism for STXs similar to that of other molluscs [36,92,93,94] but that other starfish species do not exhibit. Currently, however, the depuration and uptake kinetics of STXs in sunstars are unknown, and so would be a potential future study of interest. Previously [56], two sources of sunstar toxicity were hypothesised, either that their grazing on algal cysts produced the STXs [15] or that the presence of a symbiosis with bacteria produced the STXs [21]. As sunstars and algal cysts both occupy the benthos, the accumulation of STXs via this route is feasible. Algal cysts beds can be geographically extensive [95,96], cysts can be more toxic than vegetative cells [16] and the ingestion of algal cysts has been previously implicated in shellfish toxicity [17]. For cyst ingestion to be the principal cause of the toxin concentrations observed in *C. papposus*, the causative cyst bed/s would have to stretch around the entire UK coast and into the English Channel and be capable of producing two different toxin profiles, one of which is completely different to the profiles produced by the known UK vegetative *Alexandrium* blooms. Additionally, cyst toxin profiles have been shown to be similar to their vegetative counterparts [16]; subsequently, the resulting toxin profiles, if sunstars did ingest *Alexandrium* cysts, would likely be similar to those described in [14] and would pose the question whether the dcSTX profile came from a source other than *Alexandrium* cysts. It is also unclear why sunstars would be more susceptible to toxin accumulation via this route compared to other benthic organisms [52,56]. The cyanobacterial production of STXs is possible [7]. Although cyanobacteria are mostly associated with freshwater, there have been reports of both benthic and saltwater colonies producing STXs [19,97,98], which could explain the toxicity in a saline benthic environment; however, the same arguments for algal cysts not being the source of STXs apply to cyanobacteria, in that any cyanobacteria presence would have to be geographically extensive and it would be unclear why the uptake of STXs in sunstars is far more pronounced than in other starfish species. Due to the deeper offshore environments that sunstars often inhabit, the presence of toxins during all months of the year, the unique toxic profile and the statistical differences in the presence of STXs in sunstars, a nontraditional source should not be ruled out. The information discussed above suggests that it is feasible that sunstars accumulate STXs from somewhere other than their diet or the environment, and thus the notion that sunstars synthesise STXs internally, via a microbial symbiosis or other means, can be hypothesised. The production of the similar neurotoxin TTX in pufferfish and starfish has previously been linked to symbiotic vibrio species [24,25], so it is possible that sunstars accumulate STXs in a similar manner, which would explain the geographically widespread yet consistent toxin concentrations that were found. In conjunction, the core genes responsible for the synthesis of STXs already exist in multiple animal kingdoms [28], making the possibility of an unknown novel producer feasible. Elucidating the source could involve a multistep approach encompassing: laboratory studies to experimentally determine the accumulation and depuration kinetics of STXs in sunstars, the toxin testing of bacteria isolated and cultured from sunstars and the genetic analysis of both the microbiome and the gene clusters associated with STX production. Sediment found in sunstar environments could also be analysed; even if traditional *Alexandrium* cysts are unlikely to be the causative agent, the presence of an unknown benthic species could also be investigated.

### 3.2. Toxin Profiles

**Figure 8:**
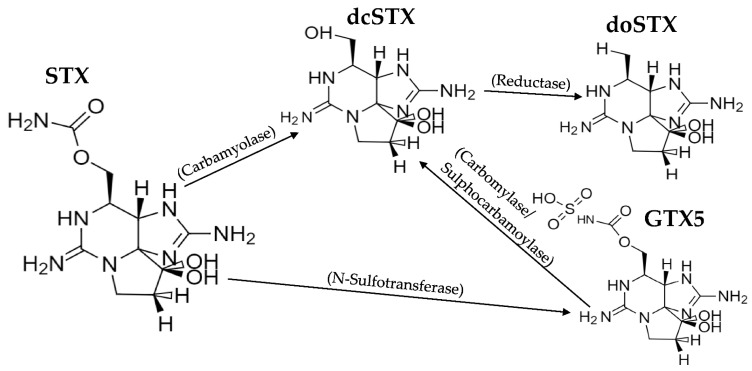
Hypothetical production route for the dcSTX profile. Enzymatic transformations from [29].

The two dominant toxic profiles are similar to those discovered previously [52,56], specifically, a high dcSTX profile from most regions and an STX- and GTX2-dominated profile from the North Yorkshire coast (Figure 2). These two distinct toxic profiles may imply two different sources of STXs, noting that whilst the high dcSTX profile is unlike any known bivalve toxin profile reported globally, the STX and GTX2 profile is similar to the profile reported from UK shellfish associated with toxin uptake from *A. minutum* [14], usually detected in the South West of the UK. The *Alexandrium* cyst beds present off the North East coast of England [99,100,101] are also of note; however, *A. catenella* (formally *A. tamerense*) was the species implicated in this region, which in the UK produces a more complex toxin profile, typically containing GTX1–5, C1/2, NEO and STX [14]. Therefore, the presence of *A. catenella* cysts does not fully explain the toxin profile prevalent off the North Yorkshire coast. The mean toxin profile for all the sunstars was heavily dominated by dcSTX in terms of the micrograms of STX equivalents per kilogram; however, in terms of the micromoles per kilogram, the relative concentrations of the lower TEF compounds doSTX and GTX5 were notably higher. Specifically, doSTX was responsible for nearly 25% of the mean molar toxin suite present, which could imply that ~25% of the dcSTX was converted, via the reduction of the hydroxyl group at R4, to the less toxic doSTX. It is currently unclear whether there was a transformation or whether the doSTX was expressed as part of the naturally produced toxic profile. In the known biosynthesis of STX, dcSTX is the last intermediate before the creation of STX, which requires the sxtL gene to facilitate the addition of the carbomyl group at R4 (Figure 1) [26,27]. The dcSTX-dominant profile could therefore be created by a source that does not possess the sxtL gene that codes for the addition of the carbomyl group at C-13 [26], or perhaps sxtL is less readily transcribed, and therefore only a small portion of the dcSTX is continued along the synthesis chain to form STX. The formation of GTX5 from dcSTX would be unusual, as GTX5 production would require the addition of a carbamoyl group at R4 and the subsequent sulfation of that carbamoyl group. It is also possible that the dcSTX profile could be the product of a series of gene- and enzyme-controlled reactions on the STX itself (Figure 8). The presence of carbomylase, for example, would convert the STX into a dcSTX, and the dcSTX could then be transformed into a doSTX via reduction at R4 (it is noted that this is not a common transformation kinetic of STXs [29]). The GTX5 present could then be a result of the sulphation of the STX at R4. Either proposed synthesis pathway would therefore require a specific set of enzymes and/or genes to be present. In order to determine the production pathway taken, molecular tools must be implemented to help discover the genes present. This will help elucidate the more likely synthesis route. For example, the presence of two distinct, geographically driven profiles infers the existence of two different genetic/enzymatic populations. In the dcSTX-dominated profile, the sulphation of STX to GTX5 could be controlled by the *sxtN* gene, whereas in the North Yorkshire profile, the sulphation of STX into GTX2 could be mediated by the *sxtSUL* gene [29]. Conversely to these hypothesis, which are not currently supported by genetic or enzymatic testing, the evidence suggests the presence of both of these profiles across a wide taxonomic range [56], which would make it unlikely that these transformations were happening in all organisms and more likely that they were representative of the toxic source itself. Therefore, if *C. papposus* can be considered as the hypothetical source of STXs in the benthos [56], then the lower concentrations exhibited by the other organisms [56] and starfish in this study could be attributed to predation on and/or trophic transfer from sunstars. Overall, however, there is currently limited evidence to determine the true nature of the toxin source that has been described in sunstars. Future work should therefore focus on the molecular analysis of the known STX-producing gene clusters at both geographic locations to isolate any obvious genetic differences in the populations, which could help elucidate the biosynthesis pathway.

### 3.3. Sunstar Physiological Analysis

Both the male and the female sunstars showed a ubiquitous toxin presence, and all the organs tested contained quantifiable concentrations of toxins. The female gonads in particular contained high concentrations of STXs, possibly highlighting their role in reproductive or larval protection. Sunstars reproduce by external fertilisation via spawning (usually in March–May in the Northern Hemisphere), in which males and females eject sperm and eggs into the water column [102]. The presence of a potent mammalian neurotoxin within the eggs and sperm in the water column has a potential ecological advantage for larval survival. There are a range of previous studies analysing the effect of STXs on different marine organisms, and the toxic effect of STXs is not limited to mammalian nervous structures. By far the most extensively researched is the effect that STXs have on molluscs, with the major examples being: reduced feeding, reduced clearance rates, reduced larval survival, reduced heart rate and shell valve closures (reviewed in [75]). In the starfish *Pisaster ochraceus*, STXs inhibited fertilisation and decreased the ability of the starfish to attach to a substrate [60], with STXs also causing mysid mortality and larval abnormalities in sea urchins [79]. STXs have also elicited negative responses in marine fish, causing neurological symptoms and mortality in multiple species [40,42] and a range of effects on rainbow trout intestinal cells [103]. STXs have also caused altered grazing strategies and reduced reproducibility in some copepods [104,105]. Previously, STXs have been proposed as a pheromone in *Alexandrium* [28] that aids in reproductive success. If STXs are used as a pheromone by sunstars, they could act as a chemical cue to initiate spawning, thus increasing the likelihood of the successful fertilisation of eggs. Conversely, previous experiments on the ‘keystone’ starfish *P. ochraceus* noted a decrease in fertilisation with an increase in the STX concentration, showing that it suppressed reproduction [60]. It should be noted that both pheromonal and larval protection could also be provided or could work in conjunction with the variety of saponins known to be produced by starfish [71,106]. In pufferfish, high concentrations of TTX were shown in the ovaries, and the inherited TTX presence in larval pufferfish acted as a deterrent for predation in juvenile pufferfish, even at low concentrations [107,108]. TTX has also been discovered in high concentrations in the eggs of the sea slug *Pleurobranchaea maculata* [109,110]. It is similarly possible that sunstars could be utilising STXs as a feeding deterrent for juvenile/larval sunstars. The presence of STXs in the digestive glands could imply that they are a potential feeding aid, with sunstars previously reported to have opened molluscs without applying much physical force [68,111] and the use of a toxic compound in predation having been proposed before [68]. STXs can illicit neurotoxicity, oxidative stress and lower metabolic capacity in bivalves [112] and could therefore potentially increase sunstar predatory success by making their prey more susceptible to their enveloping stomach. Furthermore, the presence of STXs in the skin of sunstars highlights the potential use of STXs as a chemical defense, which could work in a similar fashion to the targeted retention of STXs that has been proposed as a defense mechanism protecting some clams from predation by sea otters and siphon-nipping fish [46,113,114]. The anecdotal neurotoxicity to cats described in [68] was almost certainly caused by inherent STXs present in sunstars. Overall, there are multiple known and unknown toxicological effects of STX that act on a wide range of marine organisms; therefore, the presence of a potent neurotoxin has multiple potential ecological benefits. However, the exact role STX plays is unknown, as is whether the accumulation of STXs is passive or targeted or whether STXs are produced by sunstars themselves.

## 4. Materials and Methods

### 4.1. Sample Collection

Samples were collected from a range of locations around the coasts of England, from both inshore and offshore areas as bycatch from fishermen or washed up onshore between 16 February 2018 and 11 March 2021. There was a total of 26 individual sampling locations, separated into 12 regions, which provided a spread of data along the UK coast (Figure 2). Once collected, the samples were transported to the Weymouth laboratory where they were stored at −20 °C until required for analysis. As well as common sunstar (*Crossaster papposus*), six other species of starfish were analysed as a control group (Figure 3, Table A1), namely, sandstar (*Astropecten irregularis)*, seven-armed starfish (*Luidia cilaris*), common starfish (*Asterias rubens*), spiny starfish (*Marthasterias glacialis*), goosefoot starfish (*Anserpoda placenta*), bloody Henry starfish (*Henrica* sp.) and brittlestar (*Ophiura ophiura*).

### 4.2. Reagents and Chemicals

All solvents, reagents and chemicals were of LC–MS or HPLC grade, depending on the system-specific requirements. LC–MS grade water was produced by a MilliQ water purification system (Merck, Darmstadt, Germany). Certified reference toxins were obtained from Biotoxin Metrology, National Research Council Canada (NRCC, Halifax, NS, Canada). Toxins incorporated included GTX1–6, dcGTX2&3, dcSTX, dcNEO, NEO, STX and C1&2. Non-certified toxin standards were also received from Cawthron Natural Compounds (CNC, Nelson, New Zealand) for C3&4, dcGTX1&4 and doSTX.

### 4.3. Sample Preparation and Extraction for Toxin Analysis

Individuals of the same species from the same sampling locations were (excluding *C. papposus*) pooled together to create a representative sample. *C. papposus* samples were analysed individually to ascertain interanimal variability and whether toxicity was correlated with diameter or any physiological traits. Sunstars subjected to organ analysis were dissected, and samples of the digestive system, gonads and skin were taken. All samples were homogenised using Waring industrial blenders (Stamford, CT, USA) and IKA Ultra-Turrax homogenisers (Oxford, Oxfordshire, UK). Samples unable to be blended into a smooth paste with blenders were instead homogenised with an extraction solvent (1% acetic acid) present. Tissues were extracted using a refined method recently validated for analysis of STXs in crab, whelk and shrimp [84], specifically, a single dispersive method utilising a 1:9 sample–solvent ratio. Samples of 2.0 ± 0.1 g of homogenised tissue were extracted. Where available tissues were <2.0 ± 0.1 g, a scaled-down extraction was performed, with volumes used dependent on the volume of homogenised tissue available. Three different analytical methods were used to detect STXs in starfish samples. These were a precolumn oxidation liquid chromatography with fluorescence detection (LC–FLD) [115] method, a liquid chromatography with hydrophilic interaction chromatography coupled with tandem mass spectrometry method (HILIC–MS/MS) [116] and a LC–HRMS method (qualitative only). Where possible, samples were quantified using both the LC–FLD and HILIC–MS/MS methods; however, analysis by the HILIC–MS/MS method was prioritized due to its higher sensitivity and better analogue specificity. Therefore, all data shown in the manuscript were generated by the HILIC–MS/MS method, unless stated otherwise.

### 4.4. Sample Analysis

#### 4.4.1. Analysis of STXs by LC–FLD

**Figure 9:**
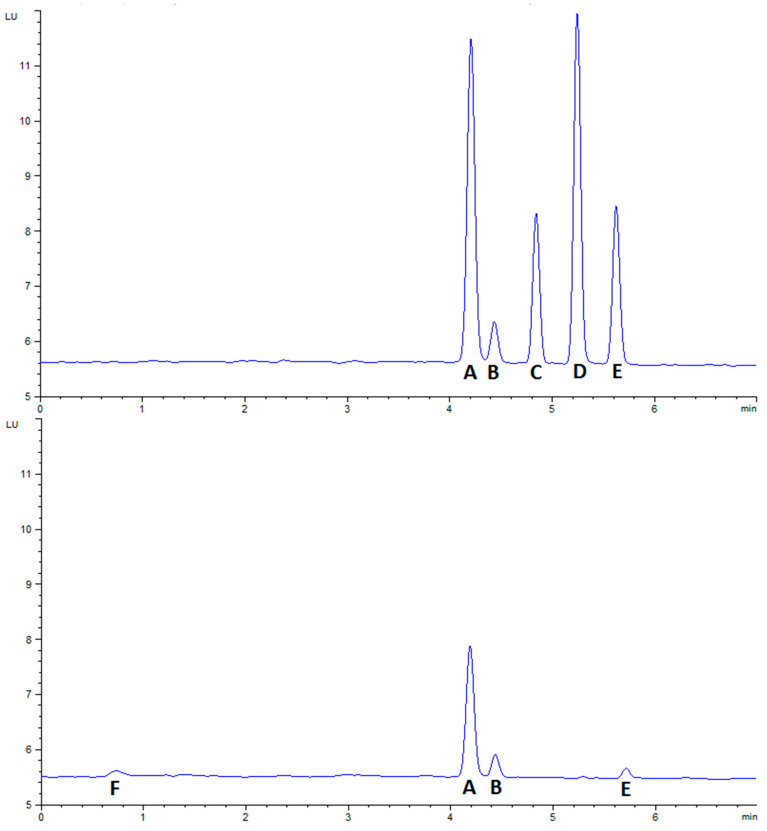
Chromatogram of LC–FLD analysis for a sunstar (**top**) and for certified standards (**bottom**). A—dcSTX (quantitative peak), B—dcSTX (qualitative peak), C—GTX2&3, D—GTX5, E—STX, F—Matrix.

Once extracted, supernatants from the centrifuged crude acetic acid extracts were subjected to a C18 solid-phase extraction step (SPE) using an automated Gilson (Dunstable, Bedfordshire, UK) ASPEC 271 system. Extracts that had been cleaned up by SPE were subsequently pH-adjusted to 6.5 ± 0.5 using 1 M NaOH and 0.1 M acetic acid and diluted to 4 mL. Analysis of samples was performed in two steps. Firstly, a semiquantitative screen (similar to that validated in [117]) was carried out to identify samples that contained any N-hydroxylated compounds, which if present would be forwarded to an ion-exchange SPE that isolated the individual fractions ready for further analysis—throughout the entire study no samples were forwarded to the ion-exchange SPE. Secondly, full quantitation of samples was achieved by the peroxide oxidation (Figure 9) of C18 SPE eluents. Analysis of unoxidised C18 SPE eluents was conducted to identify any naturally fluorescent coextractives that could interfere with chromatographic toxin peaks. LC–FLD analysis was performed on an Agilent 1200 LC system consisting of a quaternary pump, FLD, vacuum degasser, autosampler and thermostatically controlled column oven. Chromatographic separation was achieved using a Phenomenex Kinetex C18 (150 mm × 4.6 mm × 5 µm) (Torrance, CA, USA) column with a solvent gradient as per [118]. Quantitation of oxidised STXs was achieved using a six-point calibration curve prepared using certified calibrants diluted in 0.01 M acetic acid. In samples where chromatographically unresolvable epimeric pairs were present (for example, GTX2&3), the TEF of the most toxic epimer was applied (Figure 1). The LC–FLD method can quantify the epimeric pairs GTX1&4, GTX2&3, C1&2, C3&4 and dcGTX2&3, as well as the analogues GTX5, GTX6, NEO, dcNEO, dcSTX and STX.

#### 4.4.2. Analysis of STXs by HILIC–MS/MS

**Figure 10:**
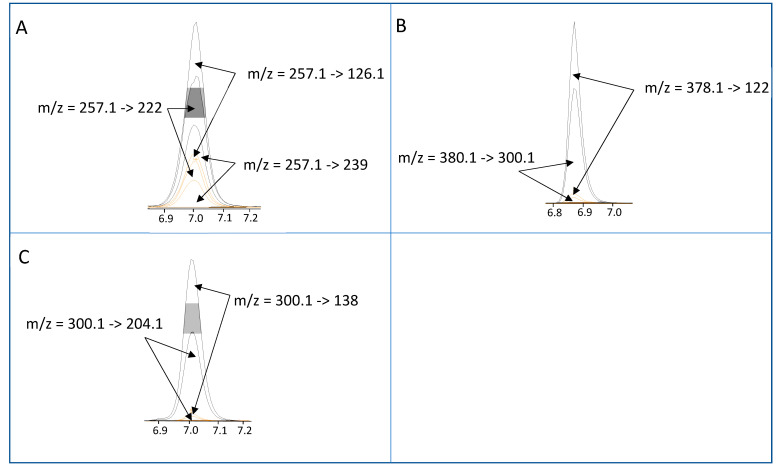
Chromatograms showing dMRMs and associated m/z transitions for (**A**) dcSTX, (**B**) GTX5 and (**C**) STX using the HILIC–MS/MS method. Grey peaks represent analytical standards and orange peaks represent sunstar sample SBS 50. X-axis = time in mins.

Crude acetic acid extracts were subjected to graphite SPE clean-up, using an automated Gilson ASPEC271 system as described in [116,119]. One hundred microlitres of SPE eluents were subsequently diluted with 300 µL of LC-MS/MS-grade acetonitrile, prior to analysis. LC-MS/MS analysis was performed using an Agilent (Manchester, UK) 6495B triple quadrupole tandem mass spectrometer, with chromatography conducted using an Agilent 1290 Infinity II UHPLC system. Chromatographic separation was achieved using either an Agilent Poroshell 120 HILICZ (150 mm × 2.1 mm × 2.7 µm) or a Waters Acquity BEH Amide (150 mm × 2.1 mm × 1.7 µm) (Elstree, Herefordshire, UK) column utilising the gradient solvent delivery method reported in [10]. Analysis of each toxin analogue was achieved using two multiple reaction monitoring (MRM) transitions [10] (Figure 10), with quantitation performed using a six-point calibration curve for each primary transition prepared using certified calibrants diluted in solvent or STX-negative, graphite SPE-cleaned and diluted mussel extract. TEFs applied were those stated in Figure 1. The HILIC–MS/MS method quantified GTX1–6, dcGTX1–4, C1-4, doSTX, dcSTX, dcNEO, NEO, and STX as well as the neurotoxin tetrodotoxin (TTX).

### 4.5. LC–HRMS Qualitative Analysis of doSTX

Qualitative assessment of doSTX presence was performed using an Agilent 1200 in tandem with an QExactive HF Orbitrap mass spectrometer. HPLC parameters were the same as those detailed in [120], specifically, analysis on a TosoHaas Amide column (250 mm × 2.1 mm × 5 µm), with separation of STXs obtained using mobile phase A (deionised water with 50 mM formic acid and 2 mM ammonium formate) and mobile phase B (100% MeCN). Analytical runs were performed at 200 µL/min using the following gradient: 90%B > 55%B over 25 min, decrease in B to 30% at 27 min and then hold at 30%B until finishing at 36 min. MS analyses were performed using a heated electrospray ionisation probe with 2500 V spray voltage and 275 °C capillary temperature. Full scan data were acquired with a resolution setting 120,000. MS/MS data were acquired using data-dependent acquisition with a resolution setting of 30,000, using stepped normalised collision energies (30, 60 and 80 eV).

### 4.6. Histological Processing and Analysis of Sunstars

**Figure 11:**
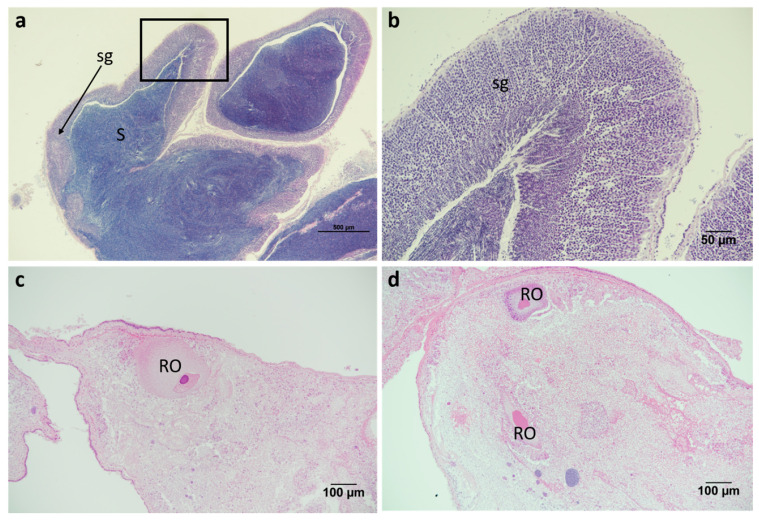
Histological examination of sunstar gonads and determination of male and female animals: (**a**) male gonads partially spawned, (**b**) higher magnification of area highlighted by box in (**a**), (**c**,**d**) female gonads spawned and spent. *sg*, spermatogenic layer; *S*, spermatozoa; *RO* residual oocytes.

Gonadal tissue from sunstars which could not be sexed through gross visual examination were dissected and fixed in Davidson’s fixative for at least 24 h. Following fixation, samples were processed in a Thermo Scientific Excelsior AS tissue processor following standard overnight routine processing schedule, where tissues were dehydrated through ethanol series, placed in xylene substitute, and then embedded in paraffin wax. Sections 3 µm thick were taken using a Leica HistoCore Multicut semiautomated rotary microtome, stained with hematoxylin and eosin, mounted and coverslipped. Sunstar gonads were sexed via light microscopy using a Nikon Eclipse E800 microscope and compared to images from [121]. Figure 11 illustrates male and female sunstar gonads.

### 4.7. Data Analysis

Data were analysed using R with ANOVAs, linear mixed-effects models and Tukey’s post hoc tests performed using the packages described in [122,123,124]. All ANOVAs, linear mixed-effects models and Tukey’s analyses were performed using log-transformed data. To make geographically driven profiles easier to analyse, sampling locations were merged into ‘regions’ which encompassed all sites from nearby locations. Analysis of toxin profiles removed all STXs < 10 µg STX eq/kg. Sunstars which were used for organ analysis were removed from the main data set and analysed separately.

## 5. Conclusions

Based on the analysis of 71 whole and 13 dissected sunstars from multiple locations along the UK coast across multiple years, this manuscript describes strong evidence for the ubiquitous presence of STXs in this species. As such, it should be considered ‘toxic’ hereafter. The sunstars sampled in this study exhibited extreme toxicities (>10,000 µg STX eq/kg) and contained statistically higher concentrations of STXs than all other starfish species tested. All the samples of sunstar skin, digestive glands and gonads (male and female) were found to contain quantifiable concentrations of STXs, with the female gonads displaying the highest total toxin concentrations (>40,000 µg STX eq/kg). The ecological role of these toxins in sunstars has yet to be elucidated; however, there are multiple proposed advantages for producing/accumulating STXs, including larval and adult defense, increasing reproductive success and use as a predation aid. The source remains unknown; however, the evidence described here hints that a traditionally described algal source may not be involved, given the ubiquitous toxin presence across a wide spatial and temporal range. Two distinct toxin profiles were confirmed, a dcSTX profile (from most UK sampling locations) and an STX and GTX2-dominated profile (from North Yorkshire). The total toxin concentrations were also shown to vary largely between the locations. Any investigation to elucidate the source should involve a multipronged strategy encompassing the following: laboratory-tank-based studies to determine the accumulation and depuration kinetics of STX in sunstars, toxin analysis of bacteria cultured from sunstars to describe any potential bacterial symbiosis and molecular analysis of both the microbiome and the gene cluster associated with the synthesis of STXs. This study and several recent manuscripts have highlighted that STXs are far more widespread than traditionally thought and that STXs possibly perform multiple unknown ecological roles in benthic marine environments around the UK coast.

## Data Availability

Data is contained within the article and Appendix A.

## Appendix A

**Table A1:** Full summary of all starfishes and brittlestars analysed. nt = not tested, nd = not detected (<1 µg STX eq/kg).

Common Name	Scientific Name	Date Sampled	Location	Region	Diameter (cm)	HILIC–MS/MS Total (µg STX eq/kg)	LC–FLD Total (µg STX eq/kg)
Common starfish	*Asterias rubens*	6 February 2018	Felixstowe	Suffolk	nt	nd	nt
Common starfish	*Asterias rubens*	6 February 2018	Felixstowe	Suffolk	nt	nd	nt
Common starfish	*Asterias rubens*	6 February 2018	Felixstowe	Suffolk	nt	nd	nt
Common starfish	*Asterias rubens*	6 February 2018	Felixstowe	Suffolk	nt	nd	1
Common starfish	*Asterias rubens*	6 February 2018	Felixstowe	Suffolk	nt	nd	4
Common starfish	*Asterias rubens*	6 February 2018	Felixstowe	Suffolk	nt	nd	nt
Common starfish	*Asterias rubens*	8 February 2018	Lowestoft	East Suffolk	nt	nd	4
Common starfish	*Asterias rubens*	8 February 2018	Lowestoft	East Suffolk	nt	nd	nd
Common starfish	*Asterias rubens*	8 February 2018	Lowestoft	East Suffolk	nt	nd	nd
Common starfish	*Asterias rubens*	8 February 2018	Lowestoft	East Suffolk	nt	nd	nd
Common starfish	*Asterias rubens*	8 February 2018	Lowestoft	East Suffolk	nt	nd	nd
Common starfish	*Asterias rubens*	8 February 2018	Lowestoft	East Suffolk	nt	nd	nd
Common starfish	*Asterias rubens*	8 February 2018	Lowestoft	East Suffolk	nt	nd	nd
Common starfish	*Asterias rubens*	8 February 2018	Lowestoft	East Suffolk	nt	nd	1
Common starfish	*Asterias rubens*	8 February 2018	Lowestoft	East Suffolk	nt	nd	nd
Common starfish	*Asterias rubens*	8 February 2018	Lowestoft	East Suffolk	nt	nd	nd
Sunstar	*Crossaster papposus*	16 February 2018	Holkham Beach	North Norfolk	nt	157	388
Common starfish	*Asterias rubens*	19 February 2018	Lulworth Banks	Dorset	nt	nd	nd
Common starfish	*Asterias rubens*	19 February 2018	Lulworth Banks	Dorset	nt	nd	nd
Common starfish	*Asterias rubens*	20 February 2018	Aldeburgh Beach	East Suffolk	nt	nd	2
Common starfish	*Asterias rubens*	20 February 2018	Aldeburgh Beach	East Suffolk	nt	nd	nd
Common starfish	*Asterias rubens*	20 February 2018	Aldeburgh Beach	East Suffolk	nt	nd	167
Common starfish	*Asterias rubens*	3 March 2018	Hunstanton Beach	North Norfolk	nt	nd	10
Common starfish	*Asterias rubens*	3 March 2018	Hunstanton Beach	North Norfolk	nt	nd	5
Common starfish	*Asterias rubens*	3 March 2018	Hunstanton Beach	North Norfolk	nt	nd	10
Common starfish	*Asterias rubens*	5 March 2018	Aldeburgh Beach	East Suffolk	nt	nd	5
Common starfish	*Asterias rubens*	5 March 2018	Aldeburgh Beach	East Suffolk	nt	nd	3
Common starfish	*Asterias rubens*	5 March 2018	Aldeburgh Beach	East Suffolk	nt	nd	7
Sunstar	*Crossaster papposus*	5 March 2018	Brancaster	North Norfolk	nt	321	781
Sunstar	*Crossaster papposus*	5 March 2018	Brancaster	North Norfolk	nt	579	1357
Sunstar	*Crossaster papposus*	5 March 2018	Wells	North Norfolk	nt	1640	4980
Sunstar	*Crossaster papposus*	5 March 2018	Hunstanton Beach	North Norfolk	nt	2727	6679
Sunstar	*Crossaster papposus*	5 March 2018	Hunstanton Beach	North Norfolk	nt	2991	4778
Sunstar	*Crossaster papposus*	5 March 2018	Wells	North Norfolk	nt	3543	8489
Sunstar	*Crossaster papposus*	5 March 2018	Wells	North Norfolk	nt	5108	16,513
Sunstar	*Crossaster papposus*	5 March 2018	Brancaster	North Norfolk	nt	nd	13,237
Common starfish	*Asterias rubens*	6 March 2018	Lincolnshire coast	Lincolnshire	nt	nd	4
Common starfish	*Asterias rubens*	6 March 2018	Lincolnshire coast	Lincolnshire	nt	nd	4
Common starfish	*Asterias rubens*	6 March 2018	Lincolnshire coast	Lincolnshire	nt	nd	1
Sunstar	*Crossaster papposus*	6 March 2018	Lincolnshire coast	Lincolnshire	nt	3847	9058
Sunstar	*Crossaster papposus*	6 March 2018	Lincolnshire coast	Lincolnshire	nt	3292	10,993
Sunstar	*Crossaster papposus*	6 March 2018	Lincolnshire coast	Lincolnshire	nt	3343	5746
Common starfish	*Asterias rubens*	6 March 2018	Ramsgate beach	Kent	nt	nd	nt
Common starfish	*Asterias rubens*	6 March 2018	Ramsgate beach	Kent	nt	2	nt
Common starfish	*Asterias rubens*	6 March 2018	Ramsgate beach	Kent	nt	nd	nt
Common starfish	*Asterias rubens*	6 March 2018	Ramsgate beach	Kent	nt	nd	nt
Common starfish	*Asterias rubens*	6 March 2018	Ramsgate beach	Kent	nt	nd	nt
Common starfish	*Asterias rubens*	6 March 2018	Ramsgate beach	Kent	nt	nd	nt
Common starfish	*Asterias rubens*	6 March 2018	Ramsgate beach	Kent	nt	nd	nt
Common starfish	*Asterias rubens*	6 March 2018	Ramsgate beach	Kent	nt	nd	nt
Common starfish	*Asterias rubens*	6 March 2018	Ramsgate beach	Kent	nt	nd	nt
Common starfish	*Asterias rubens*	6 March 2018	Ramsgate beach	Kent	nt	nd	nt
Common starfish	*Asterias rubens*	6 March 2018	Ramsgate beach	Kent	nt	3	nt
Common starfish	*Asterias rubens*	6 March 2018	Ramsgate beach	Kent	nt	4	nt
Sunstar	*Crossaster papposus*	6 March 2018	Ramsgate beach	Kent	nt	1317	7061
Common starfish	*Asterias rubens*	6 March 2018	Ramsgate beach	Kent	nt	16	4
Common starfish	*Asterias rubens*	12 March 2018	Felixstowe	Suffolk	nt	nd	nd
Common starfish	*Asterias rubens*	12 March 2018	Felixstowe	Suffolk	nt	nd	7
Common starfish	*Asterias rubens*	12 March 2018	Felixstowe	Suffolk	nt	nd	5
Spiny starfish	*Marthasterias glacialis*	19 September 2019	Cornwall—south of St.Austell	South Cornwall	nt	nd	nd
Common starfish	*Asterias rubens*	14 October 2019	Brixham	South Devon	nt	nd	nd
Common starfish	*Asterias rubens*	24 October 2019	South of Lyme Regis	Dorset	nt	nd	nd
Brittlestar	*Ophiura ophiura*	25 October 2019	South of Lyme Regis	Dorset	nt	nd	nd
Common starfish	*Asterias rubens*	14 November 2019	Brighton	Brighton	nt	nd	nd
Common starfish	*Asterias rubens*	6 December 2019	Plymouth	South Devon	nt	nd	nd
Common starfish	*Asterias rubens*	17 December 2019	Plymouth	South Devon	nt	nd	nd
Spiny starfish	*Marthasterias glacialis*	17 December 2019	Plymouth	South Devon	nt	4	22
Common starfish	*Asterias rubens*	17 December 2019	East of Whitby	North Yorkshire	nt	89	48
Common starfish	*Asterias rubens*	17 December 2019	East of Whitby	North Yorkshire	nt	8	14
Spiny starfish	*Marthasterias glacialis*	17 December 2019	East of Whitby	North Yorkshire	nt	4	8
Sunstar	*Crossaster papposus*	17 December 2019	East of Whitby	North Yorkshire	nt	360	686
Sunstar	*Crossaster papposus*	17 December 2019	East of Whitby	North Yorkshire	nt	564	909
Sunstar	*Crossaster papposus*	17 December 2019	East of Whitby	North Yorkshire	nt	880	1447
Sunstar	*Crossaster papposus*	17 December 2019	East of Whitby	North Yorkshire	nt	548	779
Sunstar	*Crossaster papposus*	17 December 2019	East of Whitby	North Yorkshire	nt	472	625
Sunstar	*Crossaster papposus*	23 January 2020	South of Brighton	Brighton	9.4	479	795
Sunstar	*Crossaster papposus*	23 January 2020	South of Brighton	Brighton	5.7	1319	1929
Sunstar	*Crossaster papposus*	27 January 2020	North Norfolk, near the Wash	North Norfolk	3.5	nt	3109
Sunstar	*Crossaster papposus*	27 January 2020	North Norfolk, near the Wash	North Norfolk	5.2	590	837
Sunstar	*Crossaster papposus*	27 January 2020	North Norfolk, near the Wash	North Norfolk	3.2	805	1429
Sunstar	*Crossaster papposus*	27 January 2020	North Norfolk, near the Wash	North Norfolk	4.5	986	1420
Sunstar	*Crossaster papposus*	27 January 2020	North Norfolk, near the Wash	North Norfolk	6.5	1034	1667
Sunstar	*Crossaster papposus*	27 January 2020	North Norfolk, near the Wash	North Norfolk	4.6	1130	1609
Sunstar	*Crossaster papposus*	27 January 2020	North Norfolk, near the Wash	North Norfolk	4	1142	2115
Sunstar	*Crossaster papposus*	27 January 2020	North Norfolk, near the Wash	North Norfolk	2.6	1188	1803
Sunstar	*Crossaster papposus*	27 January 2020	North Norfolk, near the Wash	North Norfolk	3.2	1201	1610
Sunstar	*Crossaster papposus*	27 January 2020	North Norfolk, near the Wash	North Norfolk	4	1203	1548
Sunstar	*Crossaster papposus*	27 January 2020	North Norfolk, near the Wash	North Norfolk	2.7	1242	2146
Sunstar	*Crossaster papposus*	27 January 2020	North Norfolk, near the Wash	North Norfolk	4	1386	1726
Sunstar	*Crossaster papposus*	27 January 2020	North Norfolk, near the Wash	North Norfolk	2.5	1513	2632
Sunstar	*Crossaster papposus*	27 January 2020	North Norfolk, near the Wash	North Norfolk	3.3	1612	2864
Sunstar	*Crossaster papposus*	27 January 2020	North Norfolk, near the Wash	North Norfolk	3.7	1669	2460
Sunstar	*Crossaster papposus*	27 January 2020	North Norfolk, near the Wash	North Norfolk	5.7	1672	2651
Sunstar	*Crossaster papposus*	27 January 2020	North Norfolk, near the Wash	North Norfolk	6.5	1701	2545
Sunstar	*Crossaster papposus*	27 January 2020	North Norfolk, near the Wash	North Norfolk	5.5	1738	2633
Sunstar	*Crossaster papposus*	27 January 2020	North Norfolk, near the Wash	North Norfolk	3.5	1911	2682
Sunstar	*Crossaster papposus*	27 January 2020	North Norfolk, near the Wash	North Norfolk	2.9	2011	3500
Sunstar	*Crossaster papposus*	27 January 2020	North Norfolk, near the Wash	North Norfolk	2.6	2022	3131
Sunstar	*Crossaster papposus*	27 January 2020	North Norfolk, near the Wash	North Norfolk	3.4	2075	4104
Sunstar	*Crossaster papposus*	27 January 2020	North Norfolk, near the Wash	North Norfolk	8	2128	3982
Sunstar	*Crossaster papposus*	27 January 2020	North Norfolk, near the Wash	North Norfolk	6.8	2251	3825
Sunstar	*Crossaster papposus*	27 January 2020	North Norfolk, near the Wash	North Norfolk	6.5	2455	3910
Sunstar	*Crossaster papposus*	27 January 2020	North Norfolk, near the Wash	North Norfolk	7.3	2682	3648
Sunstar	*Crossaster papposus*	27 January 2020	North Norfolk, near the Wash	North Norfolk	3.6	2776	4131
Sunstar	*Crossaster papposus*	27 January 2020	North Norfolk, near the Wash	North Norfolk	6.5	2973	4745
Sunstar	*Crossaster papposus*	27 January 2020	North Norfolk, near the Wash	North Norfolk	13	11,245	16,244
Common starfish	*Asterias rubens*	29 January 2020	Kings Lynn	North Norfolk	nt	nd	7
Goosefoot starfish	*Anseropoda placenta*	6 February 2020	Mid-central English Channel	Mid-central English Channel	nt	nd	23
Common starfish	*Asterias rubens*	6 February 2020	Mid-central English Channel	Mid-central English Channel	nt	nd	8
Brittlestar	*Ophiura ophiura*	6 February 2020	Mid-central English Channel	Mid-central English Channel	nt	121	112
Sunstar	*Crossaster papposus*	6 February 2020	Mid-central English Channel	Mid-central English Channel	9	2790	4230
Sunstar	*Crossaster papposus*	6 February 2020	Mid-central English Channel	Mid-central English Channel	12	6821	9510
Sunstar	*Crossaster papposus*	6 February 2020	Mid-central English Channel	Mid-central English Channel	9	1804	3272
Sunstar	*Crossaster papposus*	6 February 2020	Mid-central English Channel	Mid-central English Channel	12	3570	5500
Sunstar	*Crossaster papposus*	6 February 2020	Mid-central English Channel	Mid-central English Channel	14	2881	8480
Sand star	*Astropecten irregularis*	6 February 2020	Mid-central English Channel	Mid-central English Channel	nt	nd	36
Seven-armed starfish	*Luidia ciliaris*	6 February 2020	Mid-central English Channel	Mid-central English Channel	nt	nd	nd
Common starfish	*Asterias rubens*	3 March 2020	South of Bridport	Dorset	nt	nd	nd
Sunstar	*Crossaster papposus*	20 March 2020	East of Whitby	North Yorkshire	16	526	nt
Sunstar	*Crossaster papposus*	20 March 2020	East of Whitby	North Yorkshire	14	1148	nt
Sunstar	*Crossaster papposus*	20 March 2020	East of Whitby	North Yorkshire	19	984	nt
Sunstar	*Crossaster papposus*	20 March 2020	East of Whitby	North Yorkshire	17	896	nt
Sunstar	*Crossaster papposus*	20 March 2020	East of Whitby	North Yorkshire	16	933	nt
Sunstar	*Crossaster papposus*	20 March 2020	East of Whitby	North Yorkshire	14.5	492	nt
Sunstar	*Crossaster papposus*	20 March 2020	East of Whitby	North Yorkshire	17	1184	nt
Sunstar	*Crossaster papposus*	20 March 2020	East of Whitby	North Yorkshire	17	1104	nt
Sunstar	*Crossaster papposus*	20 March 2020	East of Whitby	North Yorkshire	18	930	nt
Sunstar	*Crossaster papposus*	20 March 2020	East of Whitby	North Yorkshire	15	2327	nt
Sunstar	*Crossaster papposus*	20 March 2020	East of Whitby	North Yorkshire	12	756	nt
Sunstar	*Crossaster papposus*	3 May 2020	Hunstanton Beach	North Norfolk	nt	1380	940
Sunstar	*Crossaster papposus*	3 May 2020	Hunstanton Beach	North Norfolk	nt	1607	1231
Common starfish	*Asterias rubens*	3 May 2020	Cley shingle beach (from shoreline)	North Norfolk	nt	nd	nd
Goosefoot starfish	*Anseropoda placenta*	21 July 2020	Weymouth	Dorset	nt	30	nd
Sand star	*Astropecten irregularis*	28 August 2020	South of Dartmouth	South Devon	nt	3	nt
Sand star	*Astropecten irregularis*	28 August 2020	South of Dartmouth	South Devon	nt	13	nt
Sand star	*Astropecten irregularis*	28 August 2020	South of Dartmouth	South Devon	nt	2	nt
Common starfish	*Asterias rubens*	28 August 2020	South of Dartmouth	South Devon	nt	nd	nt
Goosefoot starfish	*Anseropoda placenta*	28 August 2020	South of Dartmouth	South Devon	nt	9	nt
Bloody Henry	*Henrica oculata*	28 August 2020	South of Dartmouth	South Devon	nt	6	nt
Brittlestar	*Ophiura ophiura*	28 August 2020	South of Dartmouth	South Devon	nt	2	nt
Brittlestar	*Ophiura ophiura*	28 August 2020	South of Dartmouth	South Devon	nt	164	nt
Brittlestar	*Ophiura ophiura*	28 August 2020	South of Dartmouth	South Devon	nt	nd	nt
Seven-armed starfish	*Luidia ciliaris*	28 August 2020	South of Dartmouth	South Devon	nt	12	nt
Goosefoot starfish	*Anseropoda placenta*	28 August 2020	South of Dartmouth	South Devon	nt	4	nt
Common starfish	*Asterias rubens*	28 August 2020	South of Dartmouth	South Devon	nt	63	nt
Sunstar	*Crossaster papposus*	11 March 2021	Oban	Oban	10.5	250	270
Sunstar	*Crossaster papposus*	11 March 2021	Oban	Oban	13.5	99	125
Sunstar	*Crossaster papposus*	11 March 2021	Oban	Oban	12.5	124	134
Sunstar	*Crossaster papposus*	11 March 2021	Oban	Oban	10	425	452
Sunstar	*Crossaster papposus*	11 March 2021	Oban	Oban	13.5	291	419
Sunstar	*Crossaster papposus*	11 March 2021	Oban	Oban	13.5	307	320

**Table A2:** Overview of all starfish and brittlestars analysed. Mean toxicity, standard deviation (sd) and range are displayed in µg STX eq/kg. nd = not detected (<1 µg STX eq/kg), NA = not applicable, % > LOD = number of samples that had detectable levels of STXs above the LC–MS/MS limit of detection.

Common Name	Scientific Name	Mean Toxicity	sd	Range	n	% > *LOD*
Bloody Henry	*Henricia* spp.	6	NA	NA	1	100%
Brittlestar	*Ophiura ophiura*	96	84	nd–164	5	60%
Common starfish	*Asterias rubens*	26	35	nd–89	59	12%
Goosefoot starfish	*Anserpoda placenta*	14	14	nd–30	4	75%
Sand star	*Astropecten irregularis*	6	6	nd–13	4	75%
Seven-armed starfish	*Luidia ciliaris*	12	NA	nd–12	2	50%
Spiny starfish	*Marthasterias glacialis*	4	NA	nd–4	3	33%
Sunstar	*Crossaster papposus*	1739	1669	99–11,245	71	100%

**Table A3:** Summary of each starfish species collected in each region. Mean toxicity, standard deviation (sd) and range are displayed in µg STX eq/kg. nd = not detected (<1 µg STX eq/kg), N/A = not applicable.

Region	Species	Mean Toxicity	sd	Range	n
Brighton	Common starfish	nd	N/A	N/A	1
Brighton	Sunstar	899	594	479-1319	2
Dorset	Brittlestar	nd	N/A	N/A	1
Dorset	Common starfish	nd	N/A	N/A	3
Dorset	Goosefoot starfish	30	N/A	N/A	1
East Suffolk	Common starfish	nd	N/A	N/A	16
Mid-central English Channel	Seven-armed starfish	nd	N/A	N/A	1
Mid-central English Channel	Brittlestar	121	N/A	N/A	1
Mid-central English Channel	Common starfish	nd	N/A	N/A	1
Mid-central English Channel	Goosefoot starfish	nd	N/A	N/A	1
Mid-central English Channel	Sand star	nd	N/A	N/A	1
Mid-central English Channel	Sunstar	3573	1922	1804–6821	5
Kent	Common starfish	2	4	nd-16	13
Kent	Sunstar	1317	N/A	N/A	1
Lincolnshire	Common starfish	nd	N/A	N/A	3
Lincolnshire	Sunstar	3494	307	3292–3847	3
North Norfolk	Common starfish	nd	N/A	N/A	5
North Norfolk	Sunstar	1910	1815	157–11,245	40
North Yorkshire	Common starfish	48	57	8–89	2
North Yorkshire	Spiny starfish	4	N/A	N/A	1
North Yorkshire	Sunstar	882	466	360–2326	16
Oban	Sunstar	249	122	99–425	6
South Cornwall	Spiny starfish	nd	N/A	N/A	1
South Devon	Seven-armed starfish	12	N/A	N/A	1
South Devon	Bloody Henry	6	N/A	N/A	1
South Devon	Brittlestar	55	94	nd–164	3
South Devon	Common starfish	13	28	nd–63	5
South Devon	Goosefoot starfish	6	4	4–9	2
South Devon	Sand star	6	6	2–12	3
South Devon	Spiny starfish	4	N/A	N/A	1
Suffolk	Common starfish	nd	N/A	N/A	9

**Table A4:** Summary of individual organ analysis of sunstars. Mean toxicity, sd and range are displayed in µg STX eq/kg.

Organ	Sex	Mean Toxicity	sd	Range	n
Digestive gland	Female	2871	2115	842–5666	7
Digestive gland	Male	4672	4232	700–10,339	6
Gonad	Female	14,234	16,952	669–45,766	7
Gonad	Male	2383	2167	234–5634	6
Skin	Female	1574	1035	128–3045	7
Skin	Male	3117	2685	766–7249	6
